# A Case of Multisystem Inflammatory Syndrome in a Pediatric Patient With Acute Appendicitis

**DOI:** 10.7759/cureus.17084

**Published:** 2021-08-11

**Authors:** Sofia Ahsanuddin, Mahmud Elfituri, Eliana Diaz, Yakov Volkin

**Affiliations:** 1 Pediatrics, Icahn School of Medicine at Mount Sinai, New York, USA; 2 Pediatrics, Elmhurst Hospital Center, Icahn School of Medicine at Mount Sinai, New York, USA

**Keywords:** acute appendicitis, mis-c, pediatrics, multisystem inflammatory syndrome in children, coronary artery dilatation, sars-cov-2

## Abstract

This case report details the clinical course of a 7-year-old patient with an initial presentation of acute appendicitis, who developed symptoms highly concerning multisystem inflammatory syndrome in children (MIS-C) after appendectomy. Despite appropriate management, the patient went on to develop left main coronary artery dilatation. Given the spectrum of clinical presentations and absence of pathognomonic findings or diagnostic tests for MIS-C, it is essential to maintain a high index of suspicion for MIS-C when pediatric patients first present with nonspecific gastrointestinal symptoms and recent exposure to coronavirus disease 2019 (COVID-19). Importantly, this case illustrates that the diagnosis of MIS-C can be missed in three different ways: 1) if the patient has an absence of classic symptoms such as rash, conjunctivitis, edema, or evidence of mucocutaneous involvement on initial presentation; 2) if the patient initially has leukocytosis, instead of leukopenia (which is more prevalent in MIS-C cases), and a normal platelet count early on in the disease course; and 3) if providers confuse MIS-C for other more common postoperative causes of fever, such as atelectasis. Finally, MIS-C should still be considered part of the differential even if abdominal computer tomography (CT) findings are unremarkable for systemic inflammation. Given the potential for a rapid clinical decline in patients with MIS-C, appropriate workup should be completed in a timely manner.

## Introduction

Since the onset of the coronavirus disease 2019 (COVID-19) pandemic in late 2019, multiple reports have detailed a novel multisystem inflammatory syndrome in children (MIS-C) [1]. While the vast majority of pediatric patients infected with COVID-19 experience complete resolution of the disease, a subset of patients develop MIS-C, which occurs after the initial infection in previously asymptomatic patients [2]. Standardized definitions of MIS-C are in the process of being developed as an understanding of the disease’s pathogenesis, risk factors, and epidemiology are still evolving. The criteria defined by World Health Organization (WHO) for MIS-C include the following: 1) 0-19 years of age; 2) fever >100.4°F for three or more days; 3) clinical signs of multisystem involvement; 4) elevated markers of inflammation; 5) no other obvious microbial cause of inflammation; and 6) evidence of SARS-CoV-2 infection [3]. The Centers for Disease Control and Prevention (CDC) criteria for diagnosing MIS-C is as follows: 1) Age < 21 years; 2) fever >38°C for more than 24 hours; 3) laboratory evidence of inflammation; 4) multisystem involvement defined as having two or more organ systems involved; 5) severe illness requiring hospitalization; 6) no alternative plausible diagnoses; and 7) evidence of recent or current SARS-CoV-2 infection or exposure [3]. As illustrated by this case report, MIS-C can have a variable clinical presentation due to its multi-organ involvement. Importantly, imaging and labs can help clinch the diagnosis. However, as discussed in more detail in this case report, clinicians should exercise vigilance about excluding the diagnosis of MIS-C if initial imaging findings are fairly benign. 

## Case presentation

A previously healthy 7-year-old female of Nepali descent with no significant past medical or surgical history presented to the Emergency Department (ED) with a one-day duration of fever, nausea, and one episode of non-bilious, non-bloody emesis. The fever had a maximum temperature (T_max_) of 103°F and was managed at home with Tylenol. The patient endorsed loss of appetite and denied upper respiratory symptoms, including rhinorrhea, cough, or sore throat. Review of systems was negative for diarrhea, constipation, hematochezia, melena, or hematemesis. Notably, the patient and her father tested positive for COVID-19 four weeks prior to the patient’s presentation.

On presentation in the ED, the patient was generally well-appearing and had a fever of 100.8°F. She was tachycardic with an oxygen saturation of 98% and blood pressure of 109/68 mm Hg. On physical examination, the patient was found to have abdominal guarding and right lower quadrant tenderness with a positive Rovsing’s sign. Physical examination was negative for mucocutaneous changes, edema, rash, conjunctivitis, or any respiratory symptoms. Initial blood work revealed leukocytosis with a white blood cell (WBC) count of (16.81 x 10^3^)/mcl (74% neutrophils), platelet count of 320,000/mcl, and C-reactive protein (C-RP) of 92 mg/mL. Aspartate transaminase (AST) and alanine transaminase (ALT) were within normal limits at the time of presentation. Urinalysis did not show any abnormalities aside from trace leukocyte esterase. Abdominal ultrasound was inconclusive for appendicitis.

**Figure 1:**
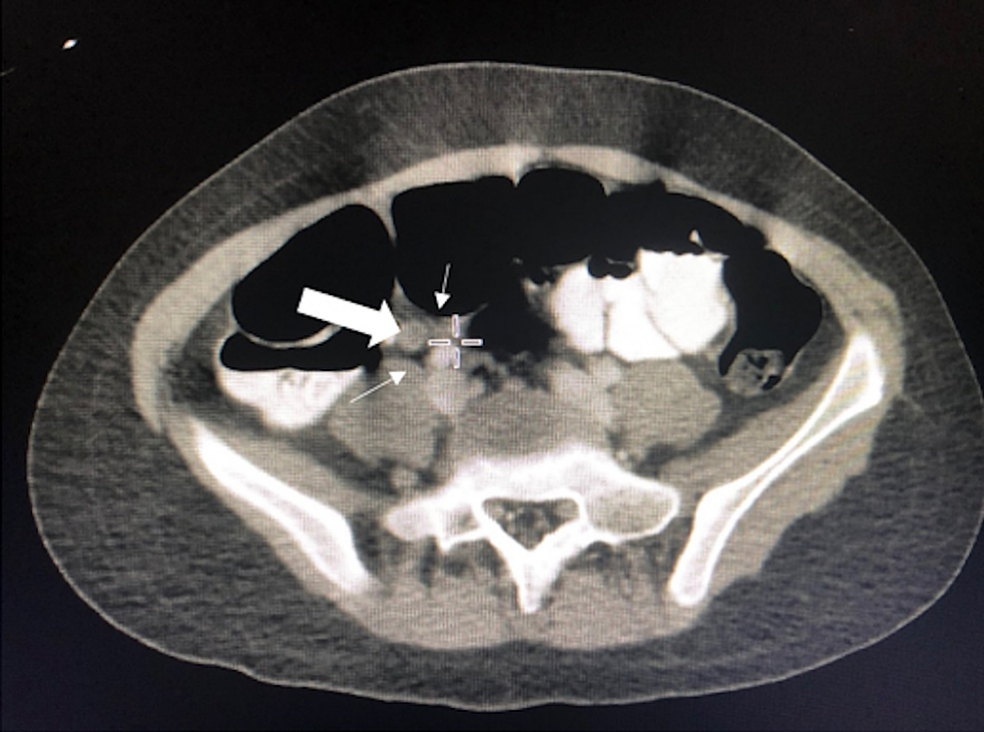
Computed tomography (CT) abdomen with contrast showing mildly inflamed appendix measuring 7-8 mm.

Given her presentation, acute appendicitis was suspected. A low-dose abdominal computed tomography (CT) scan with contrast was notable for a mildly distended, fluid-filled appendix measuring approximately 7-8 mm in maximum diameter, which was suggestive of early appendicitis without perforation, necrosis, or abscess (Figure 1). Small nonspecific mesenteric lymph nodes were noted as well. The general surgery team was consulted and the patient was started on piperacillin-tazobactam per hospital protocol for preoperative management of appendicitis. 

**Figure 2:**
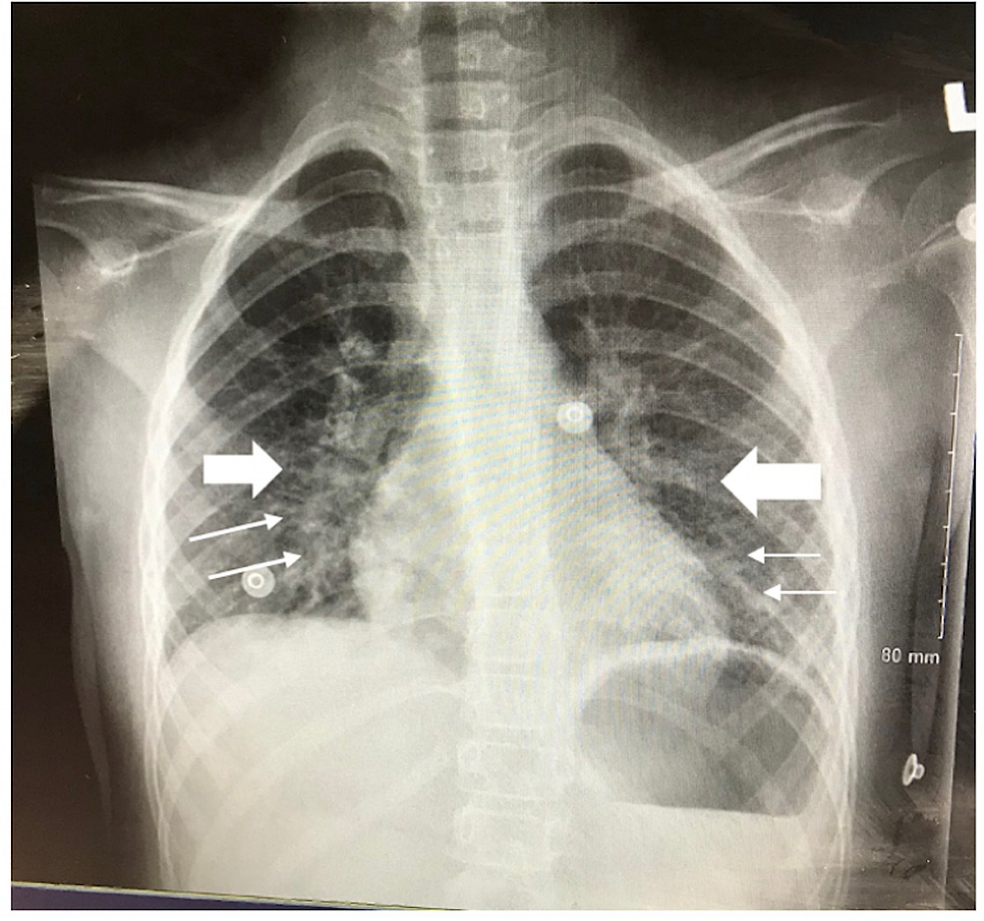
Anterior Posterior (AP) chest x-ray showing bilateral basal infiltrates concerning atelectasis.

The patient was admitted to the pediatric inpatient unit to await laparoscopic appendectomy. The pathology report from the procedure revealed a mildly inflamed appendix with no peritoneal fluid. Postoperatively, the patient remained hemodynamically stable with a temperature of 99.4°F and a blood pressure of 91/55 mm Hg with minimal postoperative pain. The patient’s emesis had resolved, she was voiding independently, and she was passing flatus with no bowel movements. However, ten hours after the surgery, she started to have a high-grade fever of 102.4°F. She became tachycardic to 140 beats per minute (bpm). Her physical examination was notable for decreased air entry on the right lower lung base, abdominal distension, and mild diffuse abdominal tenderness. Lab work was significant for leukopenia (WBC 2.7, Bands 8) with C-RP elevated to 195.4 mg/L. Chest X-ray showed bilateral basal infiltrates, a finding that prompted the surgery team to believe that the patient’s symptoms were due to postoperative atelectasis (Figure 2). The patient received two boluses of normal saline for tachycardia and she was subsequently kept on maintenance IV fluids with IV piperacillin-tazobactam and pain management as needed.

On day four, the patient’s neurological status declined precipitously; she became more irritable and uncooperative. She was hypoxic with oxygen saturation levels declining to 92%. Her maximum temperature (T_max_) was 102°F. On physical examination, she was noted to have lip swelling, periorbital swelling, and nonpurulent conjunctivitis. Given the patient’s recent exposure to COVID-19, a multisystem inflammatory syndrome in children (MIS-C) was at the top of the differential. Subsequent laboratory test reports were notable for a COVID-19 antibody index of 75.39, elevated prothrombin (PT) of 16.9 seconds, activated partial thromboplastin time (aPTT) of 41.6 seconds, D-dimer of 5,168 ng/mL, ferritin of 1881 ng/mL, and pro-brain natriuretic peptide (pro-BNP) of 2,198 pg/mL (Table 1). Her lactate dehydrogenase (LDH) was also elevated at 297 mmol/L. A complete blood count (CBC) showed a WBC count of (3.78 x 10^3^)/mcl, platelet count of 77,000/mcl, ALT of 141 U/L and AST of 137 U/L. Venous blood gas showed a pH of 7.31, bicarbonate (HCO_3_^-^) of 17 mmol/L, and an elevated lactate level of 2.9 mmol/L. Albumin was also low at 2.9 g/dL.

**Table 1:** Overview of laboratory values WBC = White blood cell count; RBC = Red blood cell count; LDH = Lactate dehydrogenase; COVID-19 IgG = coronavirus disease 2019 immunoglobulin G; C-RP = C-Reactive Protein; Pro-BNP = Pro-Brain Natriuretic Peptide; AST = Aspartate transaminase; ALT = Alanine transaminase; PT = Prothrombin time; INR = International normalized ratio; aPTT = Activated partial thromboplastin time

Laboratory Markers (Units)	Hospitalization Day 1	HospitalizationDay 3	HospitalizationDay 4	HospitalizationDay 5	Hospitalization day 6	Hospitalization day 7	Hospitalization day 8	Hospitalization day 9	Hospitalization day 10	Hospitalization day 11
WBC (x10^3^/mcl)	16.81	2.76	3.78	8.84	9.2	10.8	8.6	8.9	16.4	14.5
Neutrophils (x10^3^/mcl)	12.54	2.03	2.89	7.02	7.1	7.8	6.5	5.52	--	--
Lymphocytes (x10^3^/mcl)	3.00	0.26	0.47	1.20	1.5	2.2	2	--	--	--
Platelet count (x10^3^/mcl)	320	138	77	86	98	71	67	96	344	--
RBC (x10^3^/mcl)	4.52	3.83	3.78	3.05	3.3	3.43	3.16	3.07	--	--
LDH (U/L)	--	--	297	226	--	--	--	--	--	--
Ferritin (ng/mL)	--	--	1,881	--	1,409	1,013	314	--	--	--
C-RP (mg/L)	92.0	195.4	231.1	--	224.3	213.6	203.8	108.1	41.7	40.6
COVID-19 IgG antibodies	--	--	75.39	--	--	--	--	--	--	--
COVID-19 PCR Test	Negative	--	--	Negative	--	--	--	--	--	--
Pro-BNP (pg/mL)	--	--	2,198	13,373	1,207	891.79	--	--	--	--
Troponin (ng/mL)	--	--	<0.010	<0.010	0.05	0.10	0.04	--	--	--
AST (U/L)	--	--	137	58	66	46	47	--	--	--
ALT (U/L)	--	--	141	75	85	66	58	--	--	--
Albumin (g/dL)	--	--	2.6	2.1	2.0	1.7	2.4	2.2	2.6	2.7
Total Protein (g/dL)	--	--	4.7	6.2	6.8	5.4	5.7	5.7	6.4	6.7
HCO_3_^- ^(mmol/L)	22	17	17	17	18	18	20	26	--	--
Chloride (mmol/L)	102	112	111	115	116	114	113	114	--	--
Anion Gap (mEq/L)	10	10	12	9	6	--	--	--	--	--
PT (secs)	--	--	16.9	16.2	14.9	14.9	15.2	15.9	15.8	--
INR (ratio)	--	--	1.5	1.4	1.2	1.2	1.3	1.3	1.3	--
aPTT (secs)	--	--	41.6	53.0	60.6	51.4	43.4	27.3	28.4	--
D-dimer (ng/mL DDU)	--	--	5,168	3,518	15.82Ug/mL	12.75Ug/mL	6.22Ug/mL	8.64Ug/mL	11.74Ug/mL	3.86Ug/mL
Fibrinogen (mg/dL)	--	--	308	214	294	245	222	122	143	266
Lactate (mmol/L)	--	--	2.9	2.2	--	1.2	0.9	1.8	--	--

The patient's case was discussed with the pediatric infectious diseases and hematology teams. The patient’s bilateral infiltrates raised concern for pneumonia and vancomycin was started to cover methicillin-resistant *Staphylococcus aureus*. One unit of platelets was transfused in order to decrease the risk of bleeding. Because the patient had a platelet count below 80,000/mcl, the plan to initiate low molecular weight heparin was postponed. An electrocardiogram (ECG) showed sinus tachycardia and nonspecific ST changes. The cardiology team was consulted and a transthoracic echocardiogram showed no evidence of myocarditis, cardiac dysfunction, or coronary artery aneurysms. However, a trace of pericardial effusion was noted. Given these findings, the patient fulfilled WHO and CDC case definition criteria for MIS-C. Intravenous immunoglobulin therapy (IVIG) with a 2g/kg dose was started, and she was transferred to the pediatric intensive care unit for further management.

**Figure 3:**
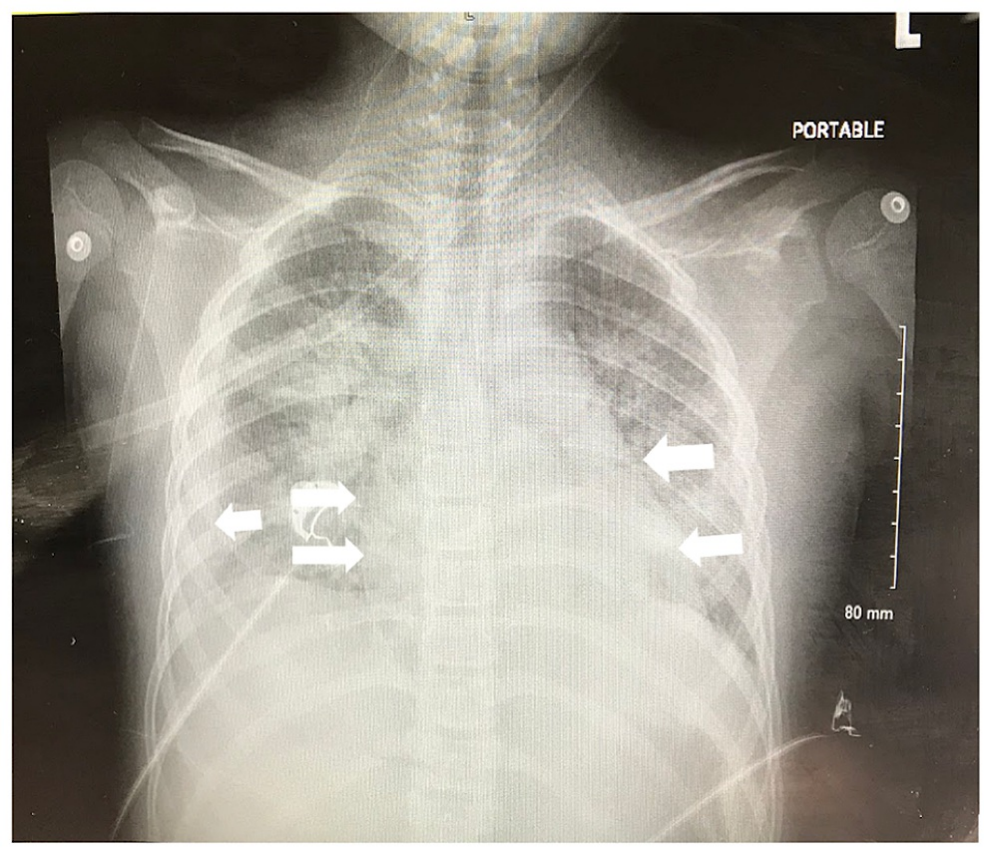
Repeat anterior posterior (AP) chest x-ray showing right-sided pleural effusion with cardiomegaly and extensive bilateral infiltrates.

On day five, the patient was afebrile and had an oxygen saturation of 85-89% on room air, which required supplemental oxygen via nasal cannula at 4 L/min. Her heart rate was 154 beats per minute (bpm) and her respiratory rate fluctuated between 30 and 40 breaths per minute. A repeat chest x-ray was performed and showed a right-sided pleural effusion with cardiomegaly and extensive bilateral infiltrates, which were thought to be attributed to infection or pulmonary edema (Figure 3). Arterial blood gas showed metabolic acidosis with a pH 7.26, carbon dioxide partial pressure (pCO_2_) of 37 mm Hg, oxygen partial pressure (pO_2_) of 179 mm Hg, and HCO_3_^-^ of 17 mmol/L. 

Due to the patient's deteriorating clinical status, she was transferred to the pediatric intensive care unit (PICU) of a tertiary care center for further management. Upon admission, she had a blood pressure of 84/50 mmHg, heart rate of 154 bpm, temperature of 99.3°F, and respiratory rate of 74 breath per minute The physical examinations were significant for increased work of breathing, decreased air entry, and fine crackles bilaterally. The patient also had periorbital edema accompanied by bilateral upper and lower extremity edema. Her laboratory test reports were significant for a platelet count of 71,000/mcl and troponin of 0.10 ng/mL. Chest x-ray showed bilateral basilar infiltrates concerning pulmonary edema versus pneumonia, for which the patient continued to receive IV vancomycin and piperacillin-tazobactam. A repeat transthoracic echocardiogram showed normal left and right ventricular function with no signs of coronary artery dilatation.

The patient was started on furosemide 10 mg twice daily and methylprednisolone 1 mg/kg for three days with significant improvement of her respiratory symptoms and oxygen requirement. A daily dose of 80 mg aspirin was also initiated. High flow nasal cannula was weaned to regular nasal cannula on the third day of her PICU stay. The following day, the patient was transferred to the pediatric inpatient floor on 2L/min oxygen. By the fifth day of her tertiary center stay, she was doing well on room air.

On day seven of the tertiary center stay, follow-up echocardiography showed an ejection fraction (EF) of 46% with decreased right ventricular (RV) function as well as a slightly dilated left main coronary artery. The same day EKG showed T wave inversions in precordial leads V4-V6. Serial troponin levels eventually trended downward. The EKG and echocardiography findings prompted concern for MIS-C myocarditis and the patient was subsequently treated with a second dose of IV immunoglobulin (IVIG). The furosemide dose was increased to 20 mg IV twice daily for left ventricular (LV) dysfunction-related volume overload.

The patient completed a seven-day course of antibiotics with negative blood cultures. After the second dose of IVIG, her platelet count recovered. The patient, however, developed anemia which was attributed to her acute illness. She was discharged home in stable condition, with close follow-up with cardiology, hematology, and her primary care pediatrician.

## Discussion

MIS-C represents a serious and potentially fatal hyperinflammatory disease with multisystem involvement in children. While the exact pathophysiologic mechanisms remain to be elucidated, it is considered to be a post-infectious phenomenon of SARS-CoV-2 infection related to an Immunoglobulin G (IgG) antibody-mediated process. Notably, pediatric patients often show prior rather than current infection with only one-third of reported MIS-C cases testing positive for SARS-CoV-2 via reverse transcription-polymerase chain reaction (RT-PCR) according to Jiang and colleagues [3]. According to Feldstein et al., dozens of published case reports on MIS-C describe a fairly nonspecific presentation with predominantly gastrointestinal (GI) symptoms such as abdominal pain, diarrhea, and vomiting in over 92% of patients [4]. These GI symptoms can potentially mimic other GI pathologies, such as pancreatitis or acute appendicitis, the latter of which was seen in our patient. Many pediatric patients with MIS-C subsequently go on to develop mucocutaneous involvement, fever, myocardial dysfunction, and cardiac abnormalities, symptoms that are reminiscent of Kawasaki’s Disease [5]. 

Importantly, according to the analysis by Feldstein et al., the median interval from initial COVID-19 infection to MIS-C onset was 25 days with a range between six and 51 days [4]. Our patient presented four weeks after the confirmed infection with the virus. Meta-analyses of case reports also demonstrate interesting demographic trends amongst affected pediatric patients; Jiang and colleagues demonstrate that male patients seem to be disproportionately affected, most patients are previously healthy with no comorbid conditions, and that an overwhelming majority of patients develop symptoms that involve four or more systems with gastrointestinal, cardiovascular, hematologic, neurologic, mucocutaneous and respiratory systems being the most affected [3]. Published reports suggest that MIS-C disproportionately affects black and Hispanic pediatric patients [6]. Additional investigation is warranted to characterize the relationship between race/ethnicity and MIS-C. 

Similar to our patient, Jackson and colleagues described a case of a 9-year-old female who presented with acute appendicitis and who eventually began to show signs of MIS-C after appendectomy [7]. However, this case was different from ours in that the imaging and pathological findings of the resected mass were far more concerning for a systemic hyperinflammatory disorder. Aslan and colleagues also published a case series of a 12-year-old female patient who had a case of appendicitis and subsequently developed pancreatitis during her hospitalization [8]. This case also had CT findings of multiple lymphadenopathies indicative of typhlitis, findings concerning a systemic inflammatory process in contrast to our own patient’s fairly benign abdominal CT findings. This suggests that there should be a high index of suspicion for MIS-C despite seemingly innocuous abdominal CT findings or even mild localized findings such as appendicitis. The diagnosis of MIS-C cannot be ruled out in the absence of lymphadenitis or generalized inflammatory changes in the abdomen. Many reports also suggest that MIS-C initially presents with leukopenia and thrombocytopenia; however, in our patient, this was not the case. Similar to the patient studied by Shenker et al., our patient’s initial laboratory test reports were notable for leukocytosis and normal platelet counts [9]. 

Mahajan and colleagues report the case of an 8-year-old patient whose only symptoms were fever and abdominal pain, the latter of which was seen in our patient in addition to emesis [10]. This suggests that it is important to keep MIS-C at the top of one’s differential even in the absence of other classic symptoms, such as rash, conjunctivitis, mucocutaneous involvement, and edema early on in the course of illness. Given the risk of rapid decompensation of patients with MIS-C, we recommend that providers maintain a high index of suspicion for MIS-C when patients present with nonspecific gastrointestinal symptoms and recent COVID-19 exposure. Additionally, the fact that our patient first presented with fever, abdominal pain and then developed other symptoms two days after her appendectomy suggests that MIS-C represents a spectrum of clinical presentations with the variable course of symptoms onset, as demonstrated in a case study by Moazzam et al. [11]. 

Finally, because the current guidelines for managing patients with MIS-C are fairly new, it is important to discuss the relative efficacy and areas for improvement of the treatment plan for our patients. Jackson et al. and Aslan et al. both reported resolution of symptoms upon initial IVIG administration. Our patient’s condition did not initially improve with IVIG infusion, but had eventually improved with administering a combination of IVIG, aspirin 80 mg, methylprednisolone 1 mg/kg, and furosemide 10 mg. Upon suspecting MIS-C myocarditis, the patient was given a second dose of IVIG and her dose of furosemide was doubled to 20 mg twice daily. Interestingly, the patient reported by Mahajan et al. recovered after the administration of anakinra and remdesivir, two agents that were not used for our patient. Further investigation is warranted to determine the efficacy of antivirals and interleukin-1 (IL-1) and interleukin-6 (IL-6) antagonists in the treatment of MIS-C.

## Conclusions

The current case demonstrates the importance of maintaining a high index of suspicion for MIS-C in pediatric patients who present with gastrointestinal symptoms and physical exam findings suggestive of acute appendicitis. Despite treatment with intravenous immunoglobulin (IVIG) within five days of symptoms onset, the patient went on to develop left main coronary artery dilatation, indicating that cardiac dysfunction could still occur later on in the disease course even with appropriate, timely management. The specific pathophysiology of the disease is still not well known. Further studies need to be conducted in order to establish management guidelines that will decrease future mortality and morbidity associated with MIS-C.
